# Development and comparative validation of multiple models for cognitive frailty in older adults residing in nursing homes

**DOI:** 10.3389/fpubh.2025.1661298

**Published:** 2025-09-15

**Authors:** Yifei Ren, Jie Ding, Jun Luo, Zhaowen Wu, Qingqing Hu, Jiajia Xu, Ting Chu

**Affiliations:** Department of Nursing, Zhejiang Chinese Medical University, Hangzhou, Zhejiang, China

**Keywords:** machine learning, cognitive frailty, nursing home, model development, older adult

## Abstract

**Objectives:**

This study aims to develop an optimal predictive model for cognitive frailty (CF) in older adults residing in nursing homes, thereby providing a scientific basis for staff to assess CF risk and implement preventive interventions.

**Methods:**

This study recruited 500 older adults from four nursing homes in Hangzhou, Zhejiang Province, between December 2024 and March 2025 as the modeling cohort. Additionally, we enrolled 112 older adults from another nursing home in Hangzhou from March to April 2025 as the external validation cohort. With 19 variables, we applied k-nearest neighbors (KNN), support vector machine (SVM), logistic regression (LR), random forest (RF), and extreme gradient boosting (XGBoost) algorithms to forecast CF. The predictive performance was assessed through multiple evaluation approaches, including ROC curve evaluation, calibration curve assessment, decision curve analysis, and various classification metrics such as accuracy, precision, recall, Brier score, and the F1-score (with *β* = 1). Furthermore, Shapley additive explanations (SHAP) value analysis was performed for the optimal model.

**Results:**

Among 500 older adults in nursing homes, 132 (26.4%) exhibited CF. Essential features included the activities of daily living (ADL), frequency of intellectual activities, and age, among others. Five models using different algorithms were developed. The SVM model demonstrated the best predictive performance, with an AUC of 0.932 on the test data. External validation confirmed its accuracy (AUC = 0.751).

**Conclusion:**

Machine learning models, particularly SVM, can effectively predict CF risk in older adults residing in nursing homes. Care facility staff can utilize personal information to assess older adults and identify high-risk individuals for CF at an early stage, providing crucial support for timely interventions and quality of life enhancement.

## Introduction

The International Institute of Nutrition and Aging and the International Association of Gerontology and Geriatrics define CF as a clinical syndrome characterized by coexisting mild cognitive impairment and physical frailty, but excludes Alzheimer’s disease and various types of dementia (1). CF represents an early clinical stage that precedes the onset of dementia (2). Unlike other cognitive impairments, the international consensus group highlights that CF stems mainly from physical conditions rather than neurodegenerative disorders. Moreover, CF may serve as a precursor to neurodegenerative processes (3). Reported prevalence rates of CF vary significantly across studies due to differences in operational definitions and population heterogeneity. Specifically, operational definitions combine physical frailty phenotypes with varying cognitive threshold, such as distinct cutoffs on the Mini-Mental State Examination and Montreal Cognitive Assessment. Population heterogeneity manifests through variations in age stratification, comorbidities, socioeconomic factors, and recruitment settings. Studies indicate that the prevalence of CF among older adults ranges from 0.72 to 30.2% in foreign populations (4, 5) and approximately 2.3 to 43.2% in domestic populations (6–8). Reportedly (6), the prevalence of CF among older adults in China is 24% in nursing homes, 24% in hospitals, and 9% in community settings. Nursing homes serve as the primary setting for older adult care, where residents often present with complex health conditions, including functional impairment, cognitive decline, and multiple chronic comorbidities (9). The confined environment and living conditions in nursing homes, coupled with limited family interaction, restrict physical activity, social engagement, and emotional support from the outside world. This increases older adults’ vulnerability to CF (10). CF elevates the risk of adverse health outcomes, including falls, functional disability, depression, prolonged hospitalization, and mortality (11, 12). Therefore, it is particularly critical to address CF among older adults in nursing homes.

Current CF prediction models, both domestically and internationally, primarily focus on community-dwelling older adults or specific disease populations. There is a critical need to develop a CF prediction model specifically for older adults in residential nursing homes. Most previous studies lacked external validation, and their practical applicability requires further verification. Currently, there is a lack of accurate predictive tools for assessing the risk of CF among older adults in residential nursing homes. The influencing factors of CF are complex, and traditional regression algorithms have limited ability to handle confounding variables, which may compromise the accuracy of predictive models. In contrast, machine learning (ML) replaces conventional predictive modeling approaches by employing computational algorithms to identify complex, non-linear interactions among variables through iterative minimization of prediction errors. It can analyze large-scale datasets and generate models with strong generalizability through ML. Park et al. (13) developed a ML-based risk assessment model for CF using data from 2,404 community-dwelling older adults in the Korean Frailty and Aging Cohort Study. This study addressed a binary classification problem, where participants exhibiting at least one physical frailty phenotype and a Mini-Mental State Examination score ≤ 24 were classified as having CF. A ML methodology incorporating recursive feature elimination and bootstrapping was employed to develop the prediction model. The model demonstrated robust predictive performance (AUC = 0.843, sensitivity = 0.751, specificity = 0.809, accuracy = 0.795), effectively identifying the risk of CF in community-dwelling older adults (13). Currently, ML is widely used in healthcare to improve the accuracy of disease prediction and diagnosis. To date, no ML-based prediction models for CF have been developed specifically for older adults in nursing homes.

Therefore, this study analyzes risk factors for CF among older adults in nursing homes and constructs ML-based risk prediction models. Furthermore, this study emphasizes model interpretability, enabling medical experts to better understand prediction outcomes, thereby providing valuable references for early prevention in nursing homes, particularly crucial in the context of accelerating population aging.

## Methods

### Study design and population

This study utilized a convenience sampling approach to recruit participants from two phases: (a) 500 older adults were enrolled from four nursing homes in Hangzhou, Zhejiang Province, between December 2024 and March 2025; (b) An additional 112 older adults were recruited from another nursing home in the same region during March to April 2025. Participants were included if the older adults were age 60 years or older, had a minimum documented residence of 3 months within the nursing home facility, maintained preserved cognitive and communication function sufficient for study procedures, and provided documented informed consent. Individuals were excluded if they had: a formal diagnosis of Alzheimer’s disease or any other form of dementia; a history of intellectual disabilities or a history of psychiatric disorders; significant communication impairments that would compromise data collection reliability; or concurrent active participation in other interventional clinical trials.

All questionnaires were collected by a single researcher during consistent time periods to ensure assessment reliability. This study adhered to the principles outlined in the Declaration of Helsinki. All participants provided written informed consent. The study protocol was approved by the Ethics Committee of Zhejiang Chinese Medical University (No. 20241129–3).

### Sample size calculation

The minimum sample size for the model was calculated based on the Events per Variable rule (14). This rule requires a sample size of at least 10 times the number of independent variables. With an estimated 10 variables to be included in this study and a reported prevalence of CF among older adults in Chinese nursing homes of approximately 24% (6), and accounting for 20% potential attrition, the required sample size was calculated as follows: 10 variables * 10 * 24% * (1 + 20%) ≈ 500 participants (15). The final sample size satisfied the empirical rule of having at least 10 events per candidate predictor variable (16).

### CF identification

Diagnosis of CF required meeting all of the following criteria based on established assessment standards and mild cognitive impairment guidelines (1): (1) Fried Phenotype score 3–5 (17); (2) education-adjusted Mini-Mental State Examination (MMSE) scores (18–20 for illiterate, 21–24 for primary education, 25–27 for secondary education or higher) (18); and (3) dementia was excluded using the Clinical Dementia Rating (CDR) scale, with a diagnosis of CF requiring a CDR score of 0.5 (19).

### Candidate variables

Based on a review of domestic and international literature and expert consensus (20–22), we included 19 predictors spanning four key domains: sociodemographic factors, health status indicators, physical function measures, and lifestyle factors.

Sociodemographic factors encompassed the following variables: age, gender (male/female), marital status (widowed, spouse alive, or never married), education level (illiterate, primary education, secondary education, or college/university and above), and post-retirement occupation (farmer, laborer, intellectual, or other). Health status indicators included: self-rated health (very poor, poor, fair, good, or very good), chronic pain (yes/no), history of falls (yes/no), depression (yes/no), and nutritional status (normal, at risk of malnutrition, or malnourished). Physical function measures comprised: ADL (normal, declined, or severely impaired), grip strength (reduced/normal), gait speed (slow/normal), body mass index – BMI (<19 kg/m^2^, 19-21 kg/m^2^, 21-23 kg/m^2^, or ≥23 kg/m^2^), and sleep duration (<6 h, 6–9 h, or >9 h). Lifestyle factors were defined as: exercise frequency (0, 1–2, or ≥3 times/week), intellectual activities frequency (0, 1–2, or ≥3 times/week), smoking history (never smoker, former smoker, or current smoker), and drinking history (never drinker, former drinker, or current drinker).

Intellectual activities encompassed cognitively stimulating pursuits such as internet use, newspaper reading, calligraphy, painting, musical instrument playing, chess, and mahjong (20). Chronic pain is characterized by persistent nociception beyond the expected duration of tissue healing, typically manifested as pain lasting at least 3 months (23). BMI is calculated by dividing an individual’s weight in kilograms by the square of their height in meters (kg/m^2^). Grip strength was measured twice for each hand using a digital dynamometer, with the highest value from the four measurements used for analysis. Gait speed was assessed by measuring the time taken to walk 6 meters at a habitual pace. Nutritional status was assessed using the Mini-Nutritional Assessment Short-Form (24). Depressive symptoms were assessed using the 5-item Geriatric Depression Scale (25). Functional status was assessed using the ADL scale (26).

### Feature selection

During data preprocessing, label encoding was applied to all categorical variables. To ensure consistent representation of CF across datasets, the modeling cohort (*n* = 500) was randomly split into training (70%, *n* = 350) and testing sets (30%, *n* = 150) using stratified sampling based on CF status. This resulted in similar minority proportions: (91/350) in the training set and (41/150) in the test set. The training set was used for model development, while the test set was used for both hyperparameter tuning and performance evaluation. Feature selection was performed using the least absolute shrinkage and selection operator (LASSO) algorithm, with the identified predictors subsequently incorporated into the predictive model. Feature selection reduces dimensionality to enhance model generalizability while mitigating overfitting risks.

### Model development

K-nearest neighbors, a non-parametric learning model, excels at capturing local patterns and is well-suited for modeling non-linear relationships in smaller sample sizes. SVM addresses complex non-linear classification problems through kernel functions. LR serves as an interpretable linear baseline for validating linear relationships. RF and XGBoost, as ensemble tree models, effectively handle high-dimensional feature interactions and collinearity, with XGBoost offering further optimized computational efficiency. Moreover, given the limited sample size of the dataset, tree-based models (RF, XGBoost) demonstrate superior resistance to overfitting compared to deep learning models. Simultaneously, as the dataset contains categorical features, tree models inherently support the processing of discrete values. Therefore, this study employs five ML algorithms—KNN, SVM, LR, RF, and XGBoost—to construct the prediction models. To mitigate overfitting, we optimized the hyperparameters using 10-fold cross-validation on the training set and evaluated the model’s generalizability with an independent test set. Additionally, 112 older adults from other nursing homes were enrolled as an external validation cohort. Finally, SHAP analysis was applied to enhance the interpretability of the optimal model.

### Model evaluation and interpretation

The ROC curve assessments were performed for all five models. To evaluate classification performance, accuracy, precision, recall, Brier score, and the F1 score were calculated for all models across training and validation datasets. Additionally, calibration curves were generated to assess the agreement between the predicted probabilities of a model and the actual observed probabilities. Finally, we used decision curve analysis to assess the clinical applicability of the models.

### Statistical analyses

This study used R 4.4.3 and Python 3.13 for statistical analysis and predictive modeling. A comprehensive two-way analysis was performed for all variable types using a generalized linear model: continuous variables were analyzed with two-way analysis of variance; binary categorical variables with binary logistic regression; ordinal categorical variables with ordinal regression; and nominal polytomous variables with multinomial logistic regression. The significance level for all hypothesis tests was set at *α* = 0.05. A *p*-value < 0.05 was considered statistically significant.

## Results

### Study population characteristics

This study enrolled 500 older adults from nursing homes, among whom 132 (26.4%) developed CF. To validate the prediction model, we recruited 112 older adults from another nursing home. CF was identified in 30 participants (26.8%). Table 1 presents the baseline characteristics of participants with and without CF. The results showed that ADL, intellectual activities frequency, age, depression, exercise frequency, education level, nutritional status, marital status, self-rated health, history of falls, BMI, post-retirement occupation, drinking history, gait speed, and chronic pain were statistically significant (*p* < 0.05).

**Table 1:** The characteristics of CF and non-CF patients in nursing homes.

Variables	Modeling set (*n* = 500)		External validation set (*n* = 112)		*P* (Cohort)	*P* (CF status)	*P* (Interaction)
	Non-CF (*n* = 368)	CF (*n* = 132)	Non-CF (*n* = 82)	CF (*n* = 30)			
Gender					0.945	0.146	0.386
Male	180 (49%)	60 (45%)	44 (54%)	12 (40%)			
Female	188 (51%)	72 (55%)	38 (46%)	18 (60%)			
Education					<0.001	<0.001	<0.001
Illiterate	9 (2%)	18 (14%)	5 (6%)	2 (7%)			
Primary education	19 (5%)	36 (27%)	25 (30%)	9 (30%)			
Secondary education	184 (50%)	45 (34%)	32 (39%)	15 (50%)			
College/University education or above	156 (42%)	33 (25%)	20 (24%)	4 (13%)			
Occupation					<0.001	<0.001	0.105
Farmer	7 (2%)	15 (11%)	6 (7%)	3 (10%)			
Laborer	62 (17%)	34 (26%)	27 (33%)	10 (33%)			
Intellectual	170 (46%)	31 (23%)	39 (48%)	9 (30%)			
Other	129 (35%)	52 (39%)	10 (12%)	8 (27%)			
Self-rated Health					0.103	<0.001	0.006
Very poor	0 (0%)	3 (2%)	1 (1%)	1 (3%)			
Poor	28 (8%)	51 (39%)	13 (16%)	8 (27%)			
Fair	88 (24%)	38 (29%)	34 (41%)	13 (43%)			
Good	194 (53%)	34 (26%)	27 (33%)	5 (17%)			
Very good	58 (16%)	6 (5%)	7 (9%)	3 (10%)			
Exercise					0.226	<0.001	0.193
0 times/week	11 (3%)	16 (12%)	5 (6%)	6 (20%)			
1–2 times/week	69 (19%)	70 (53%)	24 (29%)	11 (37%)			
3 times/week	288 (78%)	46 (35%)	53 (65%)	13 (43%)			
Intellectual activities					0.703	<0.001	0.508
0 times/week	3 (1%)	21 (16%)	3 (4%)	3 (10%)			
1–2 times/week	52 (14%)	57 (43%)	12 (15%)	16 (53%)			
3 times/week	313 (85%)	54 (41%)	67 (82%)	11 (37%)			
Smoking history					0.013	0.063	0.123
Never smoker	312 (85%)	98 (74%)	58 (71%)	21 (70%)			
Former smoker	44 (12%)	26 (20%)	20 (24%)	9 (30%)			
Current smoker	12 (3%)	8 (6%)	4 (5%)	0 (0%)			
Drinking history					0.006	0.001	0.115
Never drinker	318 (86%)	95 (72%)	57 (70%)	20 (67%)			
Former drinker	41 (11%)	33 (25%)	20 (24%)	10 (33%)			
Current drinker	9 (2%)	4 (3%)	5 (6%)	0 (0%)			
Chronic pain					0.187	<0.001	0.696
Yes	152 (41%)	82 (62%)	26 (32%)	17 (57%)			
No	216 (59%)	50 (38%)	56 (68%)	13 (43%)			
History of falls					0.972	<0.001	0.146
Yes	41 (11%)	56 (42%)	13 (16%)	10 (33%)			
No	327 (89%)	76 (58%)	69 (84%)	20 (67%)			
Sleep duration					0.624	0.824	0.008
<6 h	120 (33%)	33 (25%)	17 (21%)	12 (40%)			
6–9 h	221 (60%)	76 (58%)	50 (61%)	13 (43%)			
>9 h	27 (7%)	23 (17%)	15 (18%)	5 (17%)			
Marital					0.873	<0.001	0.631
Widowed	14 (4%)	4 (3%)	2 (2%)	1 (3%)			
Spouse alive	138 (38%)	92 (70%)	34 (41%)	19 (63%)			
Never married	216 (59%)	36 (27%)	46 (56%)	10 (33%)			
Depression					0.097	<0.001	0.054
Yes	21 (6%)	46 (35%)	12 (15%)	10 (33%)			
No	347 (94%)	86 (65%)	70 (85%)	20 (67%)			
BMI					0.139	0.030	0.468
<19 kg/m^2^	33 (9%)	15 (11%)	10 (12%)	2 (7%)			
19–21 kg/m^2^	102 (28%)	50 (38%)	19 (23%)	17 (57%)			
21–23 kg/m^2^	157 (43%)	40 (30%)	41 (50%)	9 (30%)			
≥23 kg/m^2^	76 (21%)	27 (20%)	12 (15%)	2 (7%)			
Nutrition					0.035	<0.001	0.917
Normal nutritional status	252 (68%)	48 (36%)	48 (59%)	7 (23%)			
At risk of malnutrition	106 (29%)	73 (55%)	29 (35%)	20 (67%)			
Malnourished	10 (3%)	11 (8%)	5 (6%)	3 (10%)			
ADL					0.935	<0.001	0.092
Normal	297 (81%)	26 (20%)	61 (74%)	6 (20%)			
Declined	52 (14%)	20 (15%)	11 (13%)	8 (27%)			
Severely impaired	19 (5%)	86 (65%)	10 (12%)	16 (53%)			
Grip strength					0.902	0.970	0.236
Reduced	285 (77%)	94 (71%)	58 (71%)	23 (77%)			
Normal	83 (23%)	38 (29%)	24 (29%)	7 (23%)			
Gait speed					0.581	0.003	<0.001
Slow	237 (64%)	95 (72%)	42 (51%)	23 (77%)			
Normal	131 (36%)	37 (28%)	40 (49%)	7 (23%)			
Age	85 (80, 90)	90 (87, 91.25)	86.5 (82.25, 89)	91 (88.25, 92)	0.071	<0.001	0.963

BMI, body mass index (calculated as weight in kilograms divided by height in meters squared); ADL, activities of daily living; CF, cognitive frailty; non-CF, non-cognitive frailty; *P* (Cohort), *p*-value between cohorts; P (CF Status), p value between CF states; *P* (Interaction), *p*-value for interaction.

Most disparities between CF and non-CF groups were consistent across modeling and validation sets. Notable interactions (*p* < 0.01) occurred in education level, self-rated health, sleep duration, and gait speed, suggesting cohort-specific effects in these domains.

### Feature selection

**Figure 1:**
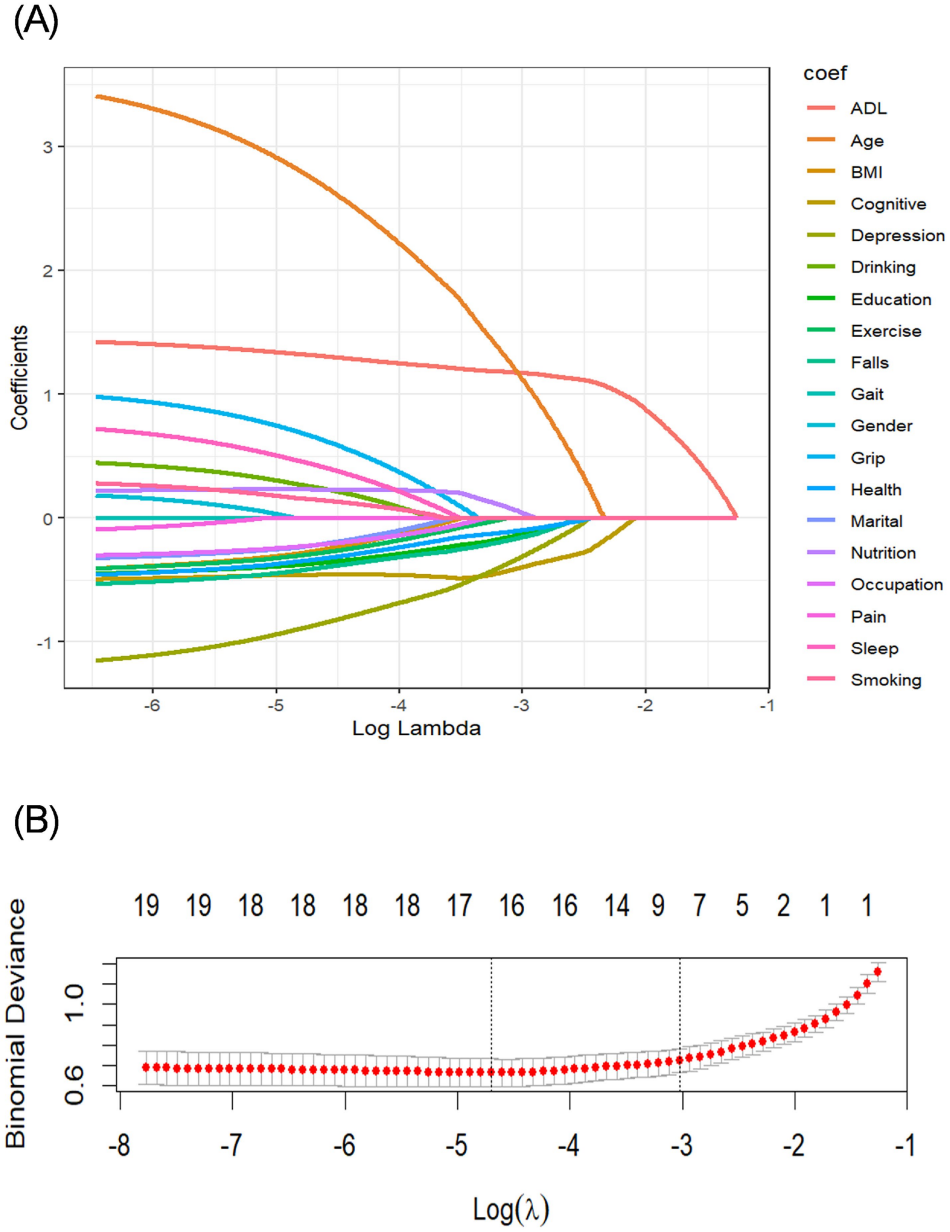
Screening variables based on LASSO regression. **(A)** Path diagram of the variable regression coefficient. **(B)** Cross-validation plot of the LASSO regression analysis. LASSO, least absolute shrinkage and selection operator.

This study included a total of 19 predictors. We used LASSO regression 10-fold cross-validation to select significant predictors of CF of the older adult in nursing homes (Figure 1). Ten feature variables were selected for the model, including ADL, frequency of intellectual activities, age, depression status, physical activity frequency, education level, nutritional status, marital status, self-rated health status, and history of fall.

### Model performance

**Figure 2:**
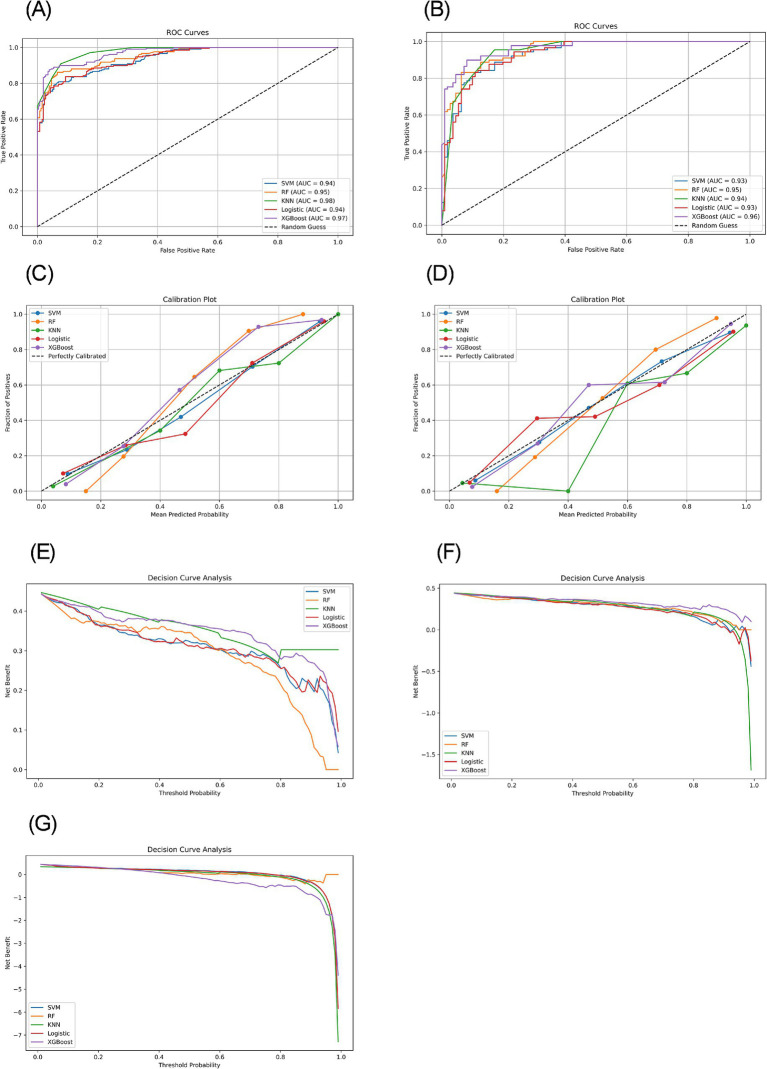
ROC curves, calibration plots, and decision curves based on different models in the training and testing sets. **(A)** ROC curves of the training set. **(B)** ROC curves of the testing set. **(C)** Calibration plots of the training set. **(D)** Calibration plots of the testing set. **(E)** Decision curves of the training set. **(F)** Decision curves of the testing set. **(G)** Decision curves of the external validation set.

**Figure 3:**
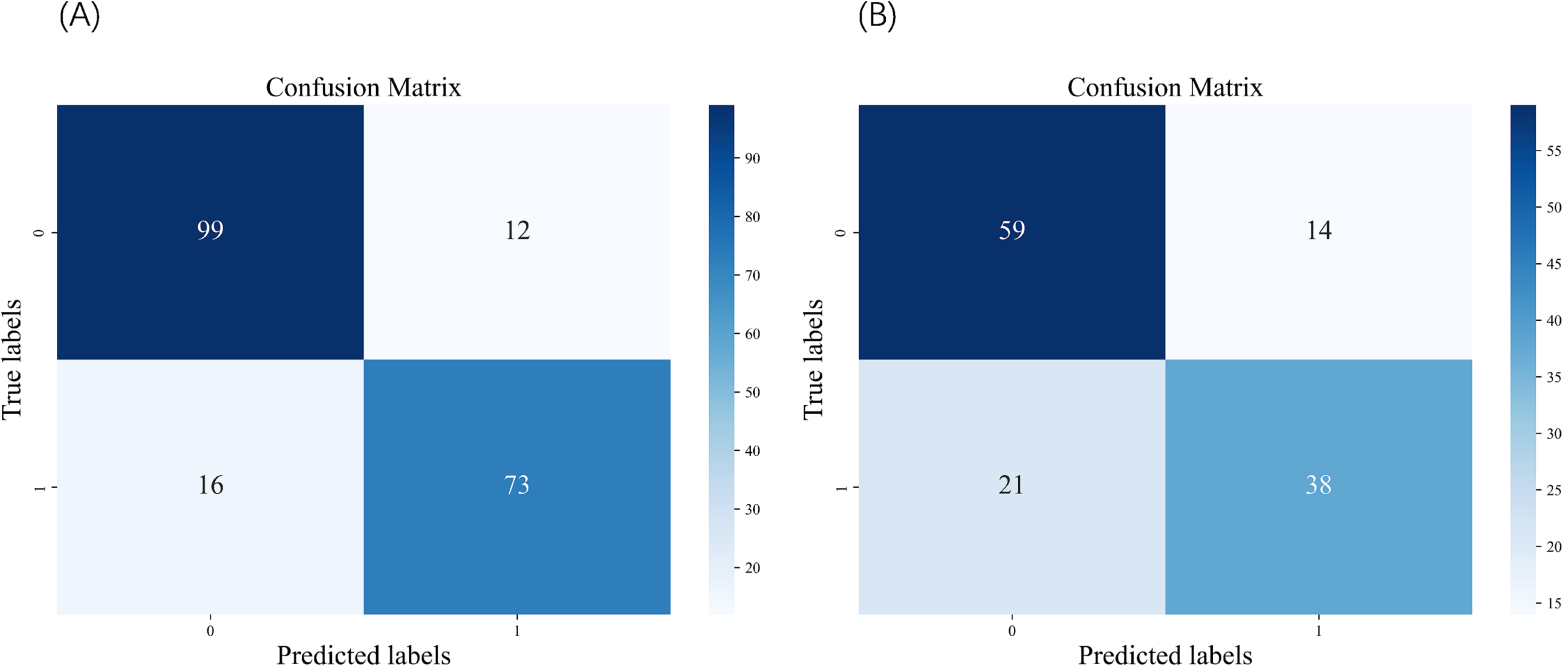
Confusion matrix of the SVM. **(A)** Confusion matrix of the test set. **(B)** Confusion matrix of the external validation set.

We constructed five different ML models, including SVM, RF, KNN, LR, and XGBoost, and evaluated their performance to predict CF. Table 2 details the performance metrics of all five models. KNN exhibited substantial degradation from train to test in accuracy (0.916 → 0.885), precision (0.905 → 0.817), F1-score (0.907 → 0.881), and AUC (0.976 → 0.945), despite recall improvement (0.909 → 0.955) (Figures 2A,B). XGBoost showed similar declines in accuracy (0.912 → 0.895), precision (0.952 → 0.895), F1-score (0.896 → 0.880), and AUC (0.970 → 0.964), though recall increased (0.847 → 0.865). Among test set performances, XGBoost achieved the highest AUC (0.964) and best Brier score (0.075), while KNN had the highest recall (0.955) but lowest precision (0.817). There may be overfitting in KNN and XGBoost. RF demonstrated balanced metrics (accuracy: 0.865, precision: 0.852, recall: 0.843, F1: 0.847, AUC: 0.951, Brier: 0.102). RF maintained minimal AUC reduction (0.953 → 0.951) with consistent performance across all metrics. LR showed exceptional stability with near-identical accuracy (0.863 → 0.860) and F1-score (0.841 → 0.841), plus modest changes in other metrics (precision: 0.876 → 0.851, recall: 0.809 → 0.831, AUC: 0.940 → 0.932, Brier: 0.096 → 0.107). SVM exhibited controlled declines in accuracy (0.876 → 0.860), precision (0.917 → 0.859), and F1-score (0.851 → 0.839), with AUC (0.938 → 0.932) and recall (0.794 → 0.820) remaining comparable. Models without significant overfitting included RF, LR, and SVM. The results of the confusion matrix for the test set are shown in Figure 3A.

**Table 2:** Models performance by different algorithms.

Algorithm	Dataset	AUC	Accuracy	Precision	Recall	F1	Brier
SVM	Train	0.938	0.876	0.917	0.794	0.851	0.097
	Test	0.932	0.860	0.859	0.820	0.839	0.105
RF	Train	0.953	0.895	0.930	0.828	0.876	0.099
	Test	0.951	0.865	0.852	0.843	0.847	0.102
KNN	Train	0.976	0.916	0.905	0.909	0.907	0.061
	Test	0.945	0.885	0.817	0.955	0.881	0.092
Logistic	Train	0.940	0.863	0.876	0.809	0.841	0.096
	Test	0.932	0.860	0.851	0.831	0.841	0.107
XGBoost	Train	0.970	0.912	0.952	0.847	0.896	0.068
	Test	0.964	0.895	0.895	0.865	0.880	0.075

Model calibration was assessed using calibration curves (Figures 2C,D) and the Brier score (Table 2). Both RF and XGBoost demonstrate strong alignment with the ideal calibration line across most probability ranges, consistent with their relatively low test-set Brier scores (RF: 0.102; XGBoost: 0.075). SVM maintains close proximity to the ideal curve in low-to-mid probabilities but exhibits slight deviations in high-probability regions, aligning with its moderate Brier score (0.105). KNN shows significant miscalibration in low-probability zones despite achieving a competitive Brier score (0.092), suggesting that this global metric may partially mask localized inaccuracies. LR displays systematic deviations in low-to-medium probabilities and the highest Brier score (0.107), indicating pronounced calibration challenges.

The decision curve analysis for both training and testing datasets revealed distinct performance characteristics among the evaluated ML models (Figures 2E,F). The XGBoost model demonstrated superior net benefit across most threshold probabilities in both datasets, although its performance was slightly diminished in the testing set, suggesting potential overfitting. Conversely, the KNN model exhibited robust performance on the training set but a significant decline on the testing set, indicative of overfitting. The LR and SVM models showed moderate and consistent net benefits across both datasets. Notably, the RF model, which underperformed on the training set, displayed improved performance on the testing set.

### External validation

In the external validation dataset (Table 3), the XGBoost model achieved the highest AUC (0.785), but exhibited substantial declines in accuracy (0.462), precision (0.454), F1 score (0.624), and probability calibration (Brier: 0.346). In contrast, SVM maintained the highest stability across metrics (accuracy: 0.735; precision: 0.731; F1: 0.685) with the best calibration performance (Brier: 0.214). The remaining models (LR, RF, KNN) demonstrated comparatively weaker overall performance and suboptimal calibration (Brier: 0.226–0.261). Collectively, while XGBoost showed suboptimal performance in external validation, SVM displayed balanced metric outcomes and superior probability calibration. The results of the confusion matrix for the external validation set are shown in Figure 3B.

**Table 3:** Models performance on the external validation set.

Algorithm	AUC	Accuracy	Precision	Recall	F1	Brier
SVM	0.751	0.735	0.731	0.644	0.685	0.214
RF	0.747	0.621	0.546	0.898	0.679	0.251
KNN	0.719	0.674	0.648	0.593	0.619	0.261
Logistic	0.747	0.712	0.691	0.644	0.667	0.226
XGBoost	0.785	0.462	0.454	1.000	0.624	0.346

Decision curve analysis was further performed to evaluate clinical utility (Figure 2G). While XGBoost achieved the highest AUC (0.785), its precision collapse (0.454) translates to a negative net benefit in decision curve analysis beyond 30% risk thresholds. This means deploying XGBoost unmodified would cause net clinical harm – misallocating resources to false positives. Conversely, SVM’s balanced precision (0.731) and recall (0.644) sustain positive net benefit across screening-relevant thresholds (10–60%), affirming its role as the safest implementation choice.

### Interpretability analysis

**Figure 4:**
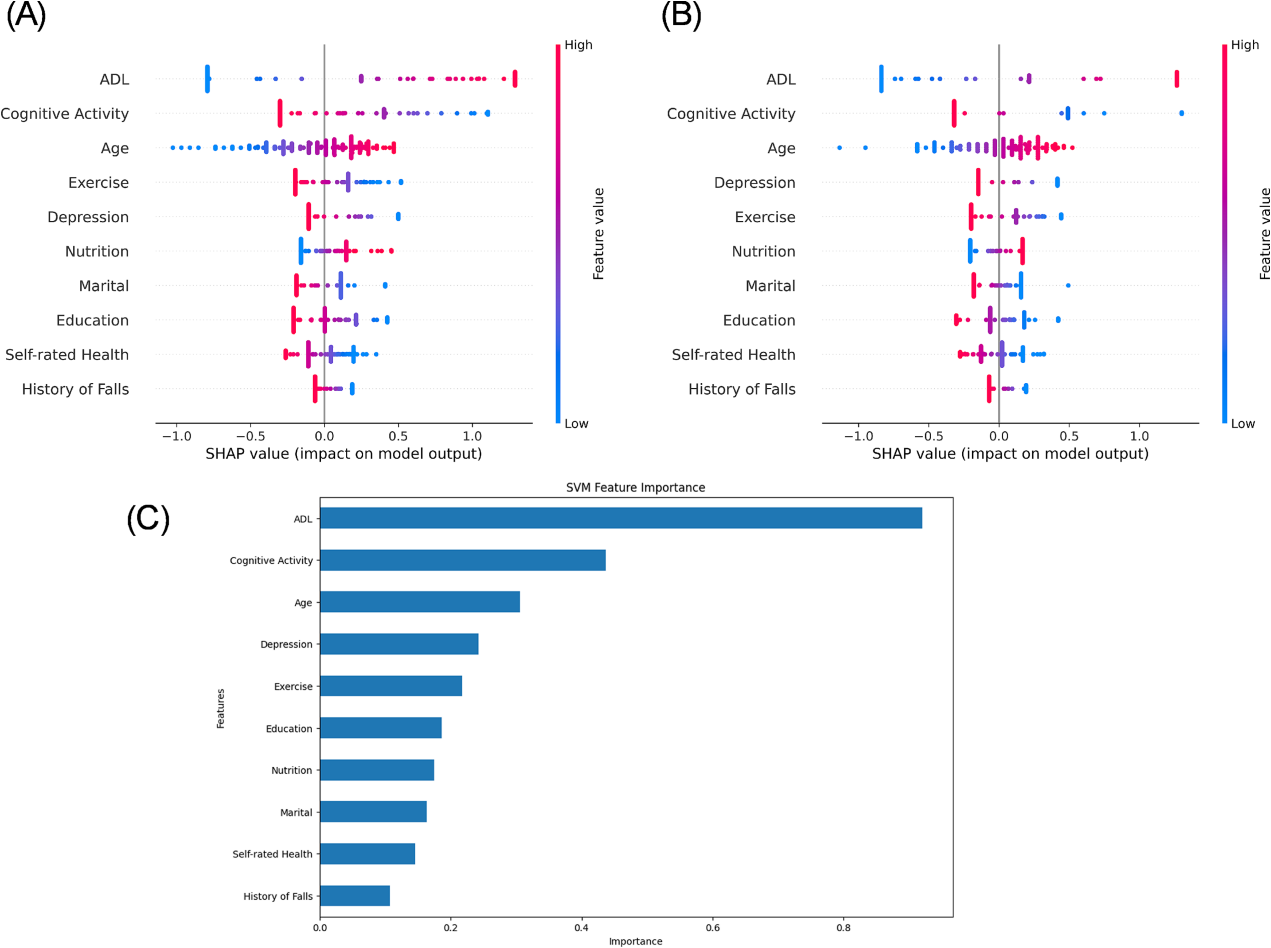
SHAP value plot for the SVM model. **(A)** SHAP analysis of the test set. **(B)** SHAP analysis of the external validation set. **(C)** Variable importance ranking plot of the test set.

The SHAP analysis of the SVM model on both the test set and the external validation set revealed consistent directional influences of key features on the predicted risk of CF (Figure 4A). Features such as ADL, intellectual activities, age, exercise, depression, and nutrition demonstrated a stable impact direction across both datasets. The external validation set SHAP plots (Figure 4B) had minor variations in order, such as depression and exercise, suggesting a consistent directional effect across datasets, but with slight variations in relative significance. Feature importance ranking of the test set (Figure 4C) confirmed ADL and intellectual activities as the most critical predictors within the model’s logic, followed by age, depression, exercise, education, nutrition, marital status, self-rated health, and history of falls. Critically, this consistency in SHAP values indicates that the model relies on a similar set of predictors and interprets their impact directionally in the same way when making predictions on both cohorts. It does not, however, imply that the underlying distributions of these features are similar between cohorts (Table 1).

## Discussion

This study developed and validated predictive models for CF among older adults in residential nursing homes using five distinct machine-learning algorithms. The SVM model demonstrated the best predictive performance, with an AUC of 0.932 on the test data. Our findings identify 10 key predictors of CF in institutionalized older adults: ADL, intellectual activities frequency, marital status, exercise frequency, depression, self-rated health, nutritional status, history of falls, age, and education level.

Our analysis reveals critical insights for deploying CF prediction models in real-world settings. While XGBoost demonstrated superior discriminative power during development (AUC: 0.964), its performance significantly declined in external validation—exhibiting substantial reductions in accuracy (0.462), precision (0.454), and F1 score (0.624) (Table 3). This contrast underscores XGBoost’s sensitivity to cohort heterogeneity, as evidenced by distribution shifts in education, nutrition, sleep duration, and functional status (Table 1). Consequently, we identify SVM as the optimal model for clinical implementation due to its balanced and stable metrics across both development (accuracy: 0.860–0.876; F1: 0.839–0.851) and external validation (accuracy: 0.735; F1: 0.685). Crucially, SVM’s metric stability directly translates to sustained positive net benefit across thresholds 10–60%, whereas XGBoost’s discriminative advantage (AUC = 0.785) is negated by precision-driven harm beyond 30% thresholds. This divergence underscores that algorithm selection for public health deployment must prioritize clinical utility over purely statistical metrics.

Two factors explain this divergence in generalizability: First, algorithmic robustness – SVM’s maximum-margin principle inherently mitigates overfitting to local feature noise, whereas XGBoost amplifies biases in underrepresented subpopulations. This proves particularly impactful given the external cohort’s higher illiteracy and malnutrition rates. Second, clinical feasibility – while all models maintained AUC > 0.7, SVM’s consistency across accuracy (0.735), precision (0.731), and F1 (0.685) minimizes implementation risk compared to XGBoost’s precision-recall tradeoffs. Rather than invalidating the models, these performance gaps highlight the necessity for context-aware deployment. In summary, SVM emerges as the most reliable model for real-world CF screening.

Despite robust performance during internal development and testing (Table 2), all models exhibited a significant decline in predictive performance on the external validation set (Table 3), highlighting a critical limitation in generalizability. This performance gap likely stems from substantial differences between cohorts in key predictors of CF—particularly education level, occupation, ADL, nutritional status, and self-rated health—as shown in Table 1. Such population heterogeneity reflects real-world clinical diversity and underscores the challenge of deploying models across settings. To bridge this gap, future research should prioritize adaptive techniques, for instance Platt scaling and other domain adaptation methods, to align models with target populations, alongside rigorous multi-site validation frameworks such as internal-external cross-validation or prospective studies to quantify real-world performance. These strategies represent the necessary steps toward reliable clinical tools.

Our study identified ADL as a significant predictor of CF in institutionalized older adults. This finding aligns with existing evidence demonstrating that individuals with ADL impairments face substantially higher risks of CF compared to those with intact functional abilities (27). The proposed pathophysiology suggests that ADL decline reduces physical activity levels, leading to decreased secretion of brain-derived neurotrophic factors and diminished cerebral blood flow – both of which may accelerate cognitive deterioration (28). The study found that nutritional status and physical activity frequency also influence CF in older adults. One study showed that participants who engaged in regular physical activity had a lower risk of CF compared to those who were inactive (29). Regular exercise and good nutritional status can effectively improve muscle strength and physical function in older adults, thereby reducing the risk of CF (30).

The CF in older adults residing in nursing homes was significantly associated with the frequency of intellectual activities, consistent with prior research findings (22). Protective effects against cognitive decline have been observed with routine intellectual engagement, such as playing mahjong or using smartphones, among community-dwelling older adults. These activities may enhance neuroplasticity and bolster cognitive reserve, thereby mitigating the progression of cognitive impairment (3).

Depression is a significant risk factor for CF in older adults, consistent with previous research (31, 32). A longitudinal study found that older adults with depression had twice the likelihood of developing CF compared to their non-depressed counterparts (33). The underlying mechanisms may involve chronic inflammation, impaired neuroplasticity, and reduced social engagement, collectively contributing to accelerated cognitive decline (34). A history of falls is recognized as a risk factor for CF. In a study by Peng et al., community-dwelling older adults with a fall history exhibited a significantly higher risk of CF compared to their non-fall counterparts (35). Potential mechanisms include traumatic brain injury, reduced physical activity, and psychological stress, all of which may contribute to impaired brain function and subsequent cognitive decline (36).

Regarding sociodemographic characteristics, marital status was identified as a significant factor influencing CF among institutionalized older adults. Zhang et al. (37) found that widowed older adults face significantly higher risks of developing CF. Furthermore, our analysis confirmed age as another critical determinant affecting CF progression in this population. Multiple longitudinal studies have established a robust association between advanced age and CF (38–40). The cumulative effect of neurodegenerative changes and progressive neuronal damage with aging contributes significantly to the gradual decline in cognitive function observed in older populations (41). Higher educational attainment is significantly associated with a reduced risk of CF in older adults. A recent study demonstrated that both education level and household per capita consumption were independently linked to CF, with higher education serving as a robust protective factor (42). Higher educational attainment is associated with greater cognitive reserve and improved access to health-related resources. Older adults with higher literacy levels frequently engage in activities such as newspaper reading and news viewing, which may enhance cognitive stimulation and reduce the risk of CF (43).

Among the 10 predictors identified, ADL, intellectual activities, nutritional status, exercise frequency, and depression represent highly actionable targets for nursing home interventions. These can be efficiently assessed using validated tools including the Barthel Index for ADL (44), Mini Nutritional Assessment-Short Form for nutrition (45), and Patient Health Questionnaire-2 for depression (46), and addressed through evidence-based protocols such as WHO-recommended chair exercises (47), targeted protein supplementation (48), and staff-facilitated group reminiscence therapy. While education, age, and marital status serve as non-modifiable risk stratification markers to identify high-risk residents for intensified monitoring, intellectual activities warrant pragmatic implementation through structured group interventions. We recommend daily 30-min music therapy sessions to enhance auditory processing, biweekly group reminiscence therapy using visual prompts to stimulate episodic memory, and weekly puzzle-based cognitive games adapted for mobility limitations (49). These low-burden strategies leverage existing staff resources while providing standardized cognitive engagement. Prioritizing these modifiable factors within implementation frameworks optimizes resource allocation in institutional settings.

Our findings provide novel insights into CF prediction and prevention strategies for older adults in residential nursing homes, with several key clinical implications. First, this multicenter study involving five nursing homes enhances the reliability of the results. Second, the model incorporates internationally validated scales, such as ADL scale, ensuring broad applicability across different care settings. Third, five distinct ML algorithms were compared, with SVM demonstrating optimal performance and superior stability. Finally, external validation (AUC = 0.751 for the SVM model) confirmed the model’s generalizability.

### Limitations of the study

This study has several limitations. First, the cross-sectional design precludes causal inferences between CF and its associated factors. Secondly, due to the significant differences in the key predictive factors of cognitive decline among different cohorts, the prediction performance of the model on the external validation set has significantly declined. Third, not all potential predictors of CF, such as biochemical markers, were included in the analysis. Fourth, label encoding may artificially establish an ordinal relationship on nominal categorical variables. Future research will develop models incorporating more comprehensive predictive features derived from longitudinal cohorts. Additionally, one-hot encoding should be applied to purely nominal features. To enhance generalizability, adaptive techniques—such as model recalibration and domain adaptation—must be prioritized for population alignment, alongside implementing a rigorous multi-site validation framework to quantify real-world performance.

## Conclusion

In this study, ADL, intellectual activities, and age emerged as the most significant predictors of CF. We developed and validated a CF prediction model for residential nursing homes using five ML algorithms, with SVM demonstrating optimal generalizability across internal and external datasets. The model maintained clinically applicable predictive performance while leveraging distributed feature dependencies. This tool may assist nursing homes in early identification of high-risk individuals, enabling targeted interventions to delay cognitive decline.

## Data Availability

The original contributions presented in the study are included in the article/supplementary material, further inquiries can be directed to the corresponding author.
